# Identification of novel SNPs in differentially expressed genes and its association with horn cancer of *Bos indicus* bullocks by next-generation sequencing

**DOI:** 10.1007/s13205-015-0351-0

**Published:** 2016-01-27

**Authors:** P. G. Koringa, S. J. Jakhesara, D. N. Rank, C. G. Joshi

**Affiliations:** Department of Animal Biotechnology, College of Veterinary Science and Animal Husbandry, Anand Agricultural University, Anand, Gujarat India

**Keywords:** SNPs, Horn cancer (HC), *Bos indicus*, *BPIFA1*, Ion Torrent PGM

## Abstract

The use of polymorphic markers like SNPs promises to provide comprehensive tool for analysing genome and identifying genomic regions that contribute to cancer phenotype. Horn cancer is the most common cancer among *Bos indicus* animals. Increased expression of some genes due to polymorphisms increases risk of HC incidence. We successfully amplified 91 SNPs located in 69 genes in 52 samples, each of HC and HN. Equimolar concentration of amplicons from 69 PCR products of each sample was pooled and subjected to sequencing using Ion Torrent PGM. Data obtained were analysed using DNASTAR software package and case control analysis using SAS software. We found SNP present in BPIFA1 gene of *B. indicus* shows association with event of HC which reflects its potential to be a genetic marker. Bioinformatic analysis to detect structural and functional impact nsSNP of BPIFA1 added another layer of confirmation to our result. We successfully identified SNP associated with HC as well as demonstrated efficient approach for limited number of SNP discovery and validation in targeted genomics regions in large number of samples combining PCR amplification and Ion Torrent PGM sequencing which suits small-scale laboratories with limited budget.

**Electronic supplementary material:**

The online version of this article (doi:10.1007/s13205-015-0351-0) contains supplementary material, which is available to authorized users.

## Introduction

Recent advances in sequencing technologies give unrivalled opportunities to explore individual genomic landscapes and identify mutations related to cancer, a complex set of diseases characterised by both environmental and genetic contributions. The use of polymorphic markers like single-nucleotide polymorphisms (SNPs) assures to provide a comprehensive tool for analysing the mammalian genome with precise identification of genomic loci contributing to the cancer phenotype. Advances in next-generation sequencing (NGS) technologies have allowed the characterisation of SNPs at genome-wide level with high densities that were previously thought to be unachievable. Thus, NGS paved the way for the fundamental understanding of mutated genes in cancer cells, affected pathways and how these data enrich our knowledge of cancer biology (Mardis and Wilson 2009).

Squamous cell carcinoma is a sporadic and highly malignant neoplasm that arises from the malpighian layer of epidermis, frequently associated with solar dermatosis. It is also known as horn cancer (HC) that involves neoplastic growth derived from proliferating horn core epithelium of predominantly mid-aged zebu bullocks affecting 1 % of cattle population (Naik et al. 1969) and causing huge economic losses due to mortality as well as reduced draft capacity as a result of prolonged morbidity. Castrated male animals, i.e. bullocks make up 95 % of the affected animals and cows 5 %, whereas it is very rare in bulls, buffaloes, sheep and goats (Chattopadhyay et al. 1982; Damodaran et al. 1979; Gupta et al. 1980; Kulkarni 1953). The incidences of HC were found to be highest in Kankrej breed of zebu cattle followed by Gir and Malvi (Joshi et al. 2009). Poorly defined genetic landscapes, absence of specific genetic markers and lack of complete understanding of genetic basis of etiopathology pose major challenges in early diagnosis. The RNA-seq based gene expression analysis indicates altered expression of numerous genes as well as multiple pathway dysfunction in HC and significant similarity in expression profiling with human lung SCC (Koringa et al. 2013a).

The large complex genome of *Bos indicus* has hindered the development of genetic resources despite the economic importance of the species. Combined efforts of high-throughput sequencing and sophisticated bioinformatics analysis will result in identifying distinctive ‘molecular portraits’ that can be correlated with clinical behaviour in cancer may also be categorised as a ‘tumour marker’ for predicting prognosis of cancer (Harris et al. 2007). A total of 26,539,698 SNPs are distributed across 4,803,648 genes in dbSNP of *B. taurus* genome build 6.1. The genome assembly of *B. indicus* is not yet available; whole genome sequencing (WGS) of *B. indicus* is currently ongoing at our research centre. The gene expression profiling in HC of *B. indicus* animals was explored with identification of SNPs in aberrantly expressed genes in our primary RNA-seq based approach.

Identification of SNPs in cancer genome using exon capture followed by sequencing (Varela et al. 2011; Xiong et al. 2012) and complete genome sequencing were proven to be costly but valuable methods for the discovery of the genetic causes of rare and complex diseases (Gonzaga-Jauregui et al. 2012; Ng et al. 2010). The said methods suit well the large-scale experiments involving studies of thousands of genes at a time. As an alternative to the above approaches, we tested a newer approach of targeted amplification of complete genomic regions followed by next-generation sequencing of genes showing aberrant expression in our previous study for identifying (Koringa et al. 2013b) and correlating with HC. In addition, comparative SNP profiling of *B. taurus* and *B. indicus* was also done to enrich the dbSNP of Indian zebu cattle.

## Materials and methods

### Clinical samples and genomic DNA isolation

Fifty-two samples were collected from cancerous horn core mucosa in RNAlater^®^ (Sigma) from clinically affected horn of Kankrej breed bullocks during the corrective surgery from different districts of Gujarat state, India [Supplementary Table 1]. The samples were stored immediately in liquid nitrogen and transferred to the laboratory. The representative sample was also collected in 10 % formalin for histopathological examination during each sample collection. Another set of 52 blood samples was collected from corresponding aged bullocks having normal horns. All the samples were histopathologically confirmed for cellular changes using paraffin embedding technique and H & E staining. DNA was isolated using the DNeasy Blood & Tissue Kit (Qiagen Inc., Valencia, CA) according to the manufacturer’s protocol.

### Amplicon generation using PCR

To amplify the genomic region of 75 genes, which were previously reported for aberrant gene expression profile and showing 100 SNPs based on a single sample transcriptome analysis (Koringa et al. 2013b), 69 pairs of specific primers were designed using the Primer 3 program (Ye et al. 2012) from the Bos_taurus_UMD_3.1.1, GenBank Assembly ID: GCA_000003055.4. There were 14 primer pairs having more than one SNP covered in their PCR product. Primers were precisely designed to have amplicon range from 250 to 346 bp so that it can be sequenced using Ion Torrent PGM 300 bp chemistry. Primer sequences, location of SNP, gene ID, amplicon size and available dbDNP ID are shown in Supplementary Table 2. Amplification of DNA was performed by PCR in 15 µl volume containing 100 ng template DNA, 10 pmol each primer, 200 nM dNTPs, 10 mM Tris HCl (pH 9.0), 50 mM KCl and 0.5U of Taq DNA polymerase (EmeraldAmp GT PCR Master Mix, Takara, Clontech Laboratories, Inc., USA) using Thermal cycler (Eppendorf, Germany). The PCR conditions were as follows: 95 °C for 5 min, followed by 31 cycles of 95 °C for 30 s, 62 °C for 15 s, 72 °C for 15 s and a final elongation step of 72 °C for 5 min. The PCR products were electrophoresed at 80 volts in a 2 % agarose gel and stained with ethidium bromide (0.5 mg/ml). The amplified products were observed under ultra violet light (300 nm) transilluminator to confirm amplification and approximation of quantity based on intensities. The size of desired amplified product was confirmed using a 50-bp molecular weight marker (Invitrogen, USA) during agarose gel electrophoresis. All the amplicons were pooled in sample-wise manner in equimolar concentration using Nanodrop1000 spectrophotometer (Thermo Scientific, USA). Each sample, after pooling, containing equal concentration of all 69 amplicons was purified using the QIAquick Gel Extraction Kit (Qiagen Inc., USA).

### Sequencing of amplicons using Ion Torrent PGM™ sequencing system

The pooled PCR products derived from all 69 amplicons were subjected to further purification step involving the Agencourt AMPure XP DNA purification beads (Beckman Coulter Genomics GmbH, Germany) to remove primer dimers and other short fragments. The same procedure was followed in each of 52 HC and HN samples. Sample-wise pooled amplicon library concentration was estimated with Qubit2.0 using the Qubit dsDNA HS assay (Life Technologies, USA). From the concentration and average size of each amplicon library, amount of DNA fragments per microlitre was calculated and libraries were diluted to 2.8 × 10^8^ DNA molecules per microlitre prior to clonal amplification. Each sample having equimolar concentration of all 69 amplicons was ligated with different barcodes [Supplementary Table 3]. Emulsion PCR was carried out using the Ion OneTouch™ 200 Template Kit v2 (Life Technologies, USA) according to the manufacturer’s instructions. Quality and quantity of the enriched spheres were checked on the Guava easyCyte5 system (Millipore GmbH, Germany) as described in the appendix of the Ion Xpress Template Kit User Guide (Part Number 4467389 Rev. B, 05/2011). Sequencing of sample-wise pooled amplicon libraries were carried out on a 316 chip using Ion Torrent PGM sequencing system and employing Ion Sequencing 300 kit (Life Technologies, USA) according to the manufacturer’s instructions. A total of five Ion Torrent PGM sequencing runs were made accommodating 20 samples in each 316 chip [Supplementary Table 4].

### Bioinformatic and global sequence analysis

After sequencing, the individual sequence read was filtered by the Ion Torrent PGM software Torrent Suite v3.6.2 to remove low quality and polyclonal sequences. Sequences matching the PGM 3′ adaptors were also automatically trimmed. All PGM quality-approved, trimmed and filtered data were exported as .sff files. Generated sequence reads were assembled using DNASTAR SeqMan NGen 11 Version: 11.2.1.25 with default parameters and the targeted variants were detected for each of the sample of HN and HC, using SeqMan Pro Version 10.0.0 against Cow-UMD_3.1genome template as reference sequence data downloaded from DNASTAR Inc. official website. The Cow-UMD_3.1 genome template contains dbSNP, gene and sequences of 3.1 build genome of *B. taurus*. For variant detection, a minimum coverage of 20 with at least 50 % of reads representing the targeted mutation (*Q* = 20; depth = 10; *P* not ref = 50) was considered mandatory. Manual curation was done for each SNP identified in both HC and HN. Locus for which at least 40 (out of 52) samples sequenced for each condition, i.e. HC and HN was selected and considered for further analysis in Arraystar 11. Each sample was individually genotyped based on sequencing data obtained and a case–control analysis of 100 samples (fifty cases of HC versus fifty samples of HN as a controls) using a logistic regression model with case status regressed on each SNPs genotype score in the SAS statistical analysis software package using JMP Genomics 6.1 (SAS, Cary, NC, USA).

### Prediction of functional impact of nsSNP

PROVEAN (Protein Variation Effect Analyser) is a tool which predicts impact of an amino acid substitution or indel on the biological function of a protein (http://provean.jcvi.org/index.php). This algorithm allows the best balanced separation between deleterious and neutral AASs, based on a threshold. A query sequence was provided in FASTA format and score for prediction based on default threshold −2.5. The score <−2.5 indicates deleterious effect and >−2.5 as neutral (Choi et al. 2012).

### Investigation of mutant protein stability by I-Mutant 2.0

I-Mutant 2.0 (http://folding.biofold.org/cgi-bin/i-mutant2.0) is a support vector machine-based web server for the automatic prediction of protein stability changes upon single-site mutations. The RI value (Reliability Index) is computed only when the sign of stability change is predicted and evaluated from output of the support vector machine. The input FASTA sequence of protein along with the residues change was provided for analysis of DDG value (kcal/mol) (Capriotti et al. 2005). The I-Mutant based analysis was carried out at pH 1, 4, 7, 9 and 12 looking to the sensitivity of BPIFA1 protein at different pH.

### Prediction of nsSNPs’ impact on surface and solvent accessibility area (SCA) of BPIFA1 protein

Solvent accessible area (ASA) of an amino-acid is helpful for locating active sites in 3D structure of proteins. The FASTA sequence of BPIFA1 was submitted to NetSurfP server (http://www.cbs.dtu.dk/services/NetSurfP/) for predictions of solvent accessibility or surface accessibility of amino acids. This algorithm is based on *Z* score which predicts surfaces. The solvent accessibility may be buried, partially buried and exposed, i.e. low accessibility, moderate accessibility or high accessibility, respectively (Petersen et al. 2009).

SNPs found to be positively correlated with HC were further analysed using PROVEAN and I-Mutant to identify effect of variants on protein structure, biological function predictions and protein stability changes.

## Results

**Fig. 1:**
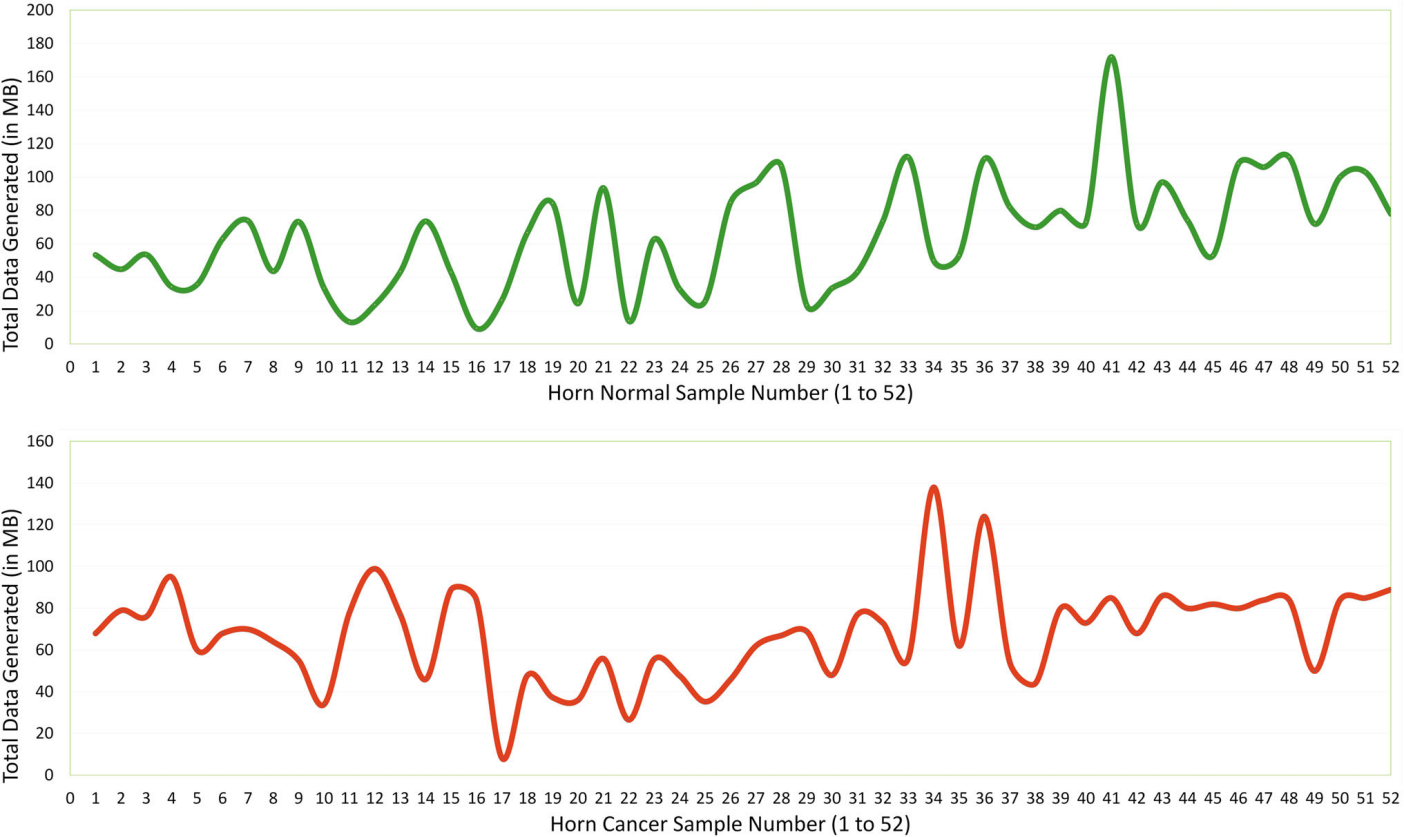
Summary of amplicon sequencing runs for each sample of HC and HN using Ion Torrent PGM sequencing system

To validate the SNPs identified in our previous study as well as to investigate the suitability of amplicon-based sequencing in identifying mutations in marginally small number of samples using Ion Torrent PGM, we successfully amplified the 91 SNPs located in 69 genes out of 100 targeted SNPs across the 75 genes in 52 samples of each HC and HN (91 % coverage of targeted SNPs). Equal amounts of DNA from 69 PCR products were pooled and subjected to Ion Torrent PGM sequencing system using 316 chip and 300 bp chemistry. Amplification of each individual locus for each sample before pooling, instead of multiplex PCR, ensured that the 69 pairs of primers were equally represented in the final product for sequencing. This is important, as different amount of DNA generated for each amplicon in multiplex PCR may introduce bias in the final representation of samples for NGS. Ion Torrent PGM constitutes a new type of next-generation benchtop sequencing platform which offers small size, low acquisition price and fast turnaround time. Here, we carried out five Ion Torrent PGM runs to analyse SNPs among two groups of 52 samples each. No obvious differences in data output between both the groups could be observed. A total data output for each sample after post run analysis using Torrent Suite Software Version 3.6.2 is given in Fig. 1 and Supplementary Table 1.

Sample-wise data output varies from 8.3 to 172 MB. Single-nucleotide polymorphism with quality score greater than 20 and minimum coverage 20 was analysed using DNASTAR software package. We also created a bedGraph format file for all the SNPs identified for easy upload to the UCSC genome browser for viewing the SNPs along with the genome annotations. Identified SNPs with reference position, dbSNP ID, feature name, protein change, base calling, frequency and genotype for each of HC and HN sample are given in Supplementary Table 2. We found 11 and 10 ‘reference’; 57 and 62 ‘homozygous variant’; 23 and 19 ‘heterozygous reference’; no “heterozygous nor reference’ genotype in HC and HN, respectively. Reference genotype indicates that SNP reported in our previous study (based on single sample) was not correct. Fifty-six SNPs commonly showing homozygous variant genotype in both conditions can be consider *B. indicus* breed specific SNPs. Sixteen SNPs commonly showing heterozygous reference genotypes in both conditions may be due to exotic inheritance in *B. indicus* cattle as the sampling was done based on phenotypes only. The list of SNPs showing differential genotypes is given in table below with ‘NegLog10_ProbAllele’ estimated using case–control study in Table 1.

**Table 1:** List of SNPs showing different genotypes between HC and HN

SNP ID	Ref pos	dbSNP ID	Feature name	NegLog10_ProbAllele	Ref seq	HC-called seq			HN-called seq			Protein change	HC	HN
AC_000170	63251805	136870681	BPIFA1	3.398	T	T|C	0.5	0.5	T|C	0.2	0.8	F189S	Het. Ref.	Hom. Vari.
AC_000163	87328340	109751733	ODAM	2.804	–	A|–	0.14	0.86	A|–	0.36	0.64	F54fs	Hom. Vari.	Het. Ref.
AC_000170	74274533	137420399	PI3	2.148	G	G|A	0.37	0.63	G|A	0.2	0.8	R172Q	Het. Ref.	Hom. Vari.
AC_000158	147024541	110653421	COL18A1	1.920	T	C		1	T|C	0.06	0.94	L750P	Hom. Vari.	Hom. Vari.
AC_000160	14700388		LMNA	1.710	A	T|A	0.36	0.64	T|A	0.21	0.79	D262E	Het. Ref.	Hom. Vari.
AC_000159	112889632	109829723	SERPINE2	0.795	C	T|C	0.39	0.61	T|C	0.3	0.7	W107.	Het. Ref.	Hom. Vari.
AC_000159	105366897	211290601	IGFBP2	0.712	T	T|C	0.77	0.23	T|C	0.67	0.33		Ref.	Het. Ref.
AC_000158	120090702	41608018	GYG1	0.541	T	T|C	0.35	0.65	T|C	0.28	0.72	S293G	Het. Ref.	Hom. Vari.
AC_000176	14615220	108978307	WFDC18	0.451	C	T|C	0.73	0.27	T|C	0.67	0.33	A48V	Ref.	Het. Ref.
AC_000180	25861437	382138331	BLA-DQB	0.253	T	T|A	0.69	0.31	T|A	0.71	0.29	E78V	Het. Ref.	Ref.
AC_000158	147024648		COL18A1	0.022	G	G|A	0.48	0.53	G|A	0.47	0.53	A786T	Het. Ref.	Hom. Vari.

Except only one SNPs present in BPIFA1 gene, all other SNPs targeted for validation through amplicon sequencing resulted into negative association with HC. In our study SNP [T → C] at position 63251805 (dBSNP ID rs136870681) identified in BPIFA1 on chromosome number 13 of *B. indicus* shows association with event of HC which reflects its potential to be a ‘genetic marker’ in HC based on a case–control analysis. SNP present in *BPIFA1* was already reported during WGS of *B. taurus* but further information about its association with any trait is not known (Liao et al. 2013).

### Analysis of functional impact of nsSNP of BPIFA1

To add layer of confirmation to the effect of nsSNP of BPIFA1 on protein function, we investigated the functional impact of amino acid substitution. This algorithm predicted only one variant F189S as a neutral with score of 3.836.

### Analysis of stability of BPIFA1 mutant protein

In order to increase overall prediction accuracy, we used I-Mutant 2.0 SVM tool for checking stability of mutant BPIFA1 protein using protein sequence at different pH. It predicted a single amino acid substitution resulting in change in protein stability with the help of regression estimator for predicting the related free energy change of protein stability (DDG values). It predicted that the BPIFA1 mutant protein F189S with value DDG < 0 and RI value =3, which indicates decreased stability at all levels of pH and temperature of 25 °C due to mutation.

### Analysis of SCA of BPIFA1

SCA is often used for calculating the transfer free energy required to move a protein from aqueous solvent to a non-polar solvent. Amino acid substitution may produce alterations in the structure of protein, especially in case of nsSNPs. Thus, it is helpful for improving prediction of protein secondary structure. In order to understand conformational changes due to amino acid substitutions, we analysed the SCA for both BPIFA1 wild and mutant sequences. The mutant were found low accessible surface area with buried class with change in RSA, ASA and Z-fit value from 0.260 → 0.264; 52.202 → 30.988 and 0.677 → 0.580 respectively.

## Discussion

In this research paper, we tested identification and validation of targeted genomic amplification of predetermined genomic locus using NGS in a marginally small number of samples using 316 chip of Ion Torrent PGM sequencing system. We performed amplification of 75 targeted regions in 96-well PCR plates at a time following common PCR protocol. We succeeded in amplifying 69 genes. As a result of utmost care taken in quantitation of each library before pooling, we could sequence all 91 SNPs located in 69 genes with at least 20 reads covering each SNP. Our analysis suggested that the amplification of a predetermined genomic regions followed by the next-generation sequencing is an effective way to identify SNPs without going for complete sequencing of the whole genome. This approach will be effective in quickly assessing the spectrum of SNPs in important genes or genomic regions affecting HC. Similar observations were also reported in other studies related to SNP discoveries in variety of diseases (Cheng et al. 2010; Elliott et al. 2012; Junemann et al. 2012; Lopez-Doriga et al. 2014). Our approach not only identify SNPs in exons, but also SNPs located in introns and up- and downstream regions, which have been recognised more important as playing important roles in gene regulation and diseases (Jaillon et al. 2008). Despite all care taken for equimolar pooling of libraries before emulsion PCR, sample-wise data output varies significantly from 8.3 to 172 MB with an average of 69 MB; the possible reason could be preferential amplification during emulsion PCR towards genomic regions having different nucleotide sequence composition as reported in many groups of scientists across various experiments (Acinas et al. 2005; Becker et al. 2000; Kanagawa 2003; McInroy et al. 2014).

We found nine commonly found ‘reference genotype’ in both conditions that represent contrasting results to that of our previous study. Since our previous identification of SNP was based on one sample in contrast to present validation on 104 samples with sequencing depth of 20 for each SNP, the result of this research is justified. We found 56 *B. indicus* specific SNPs showing ‘homozygous variant genotype’ in both conditions. So far only 42 validated SNPs have been reported at dbSNP build 130 Genome build 4.1 of ‘*B. indicus* × *B. taurus*’ at GenBank http://www.ncbi.nlm.nih.gov/projects/SNP/snp_summary.cgi?build_id=130). SNPs validated in this study will significantly enrich database and will serve as resource for array-based genome-wide association studies. India adopted strategies to introduce exotic inheritance of *B. taurus* draft purpose cattle breeds to increase overall productivity resulted into some exotic inheritance is very well represented by 17.5 % SNPs commonly showing ‘heterozygous reference’ in both conditions as the sampling for present study was based on phenotypic characters only.

A case control analysis of 52 cases of HC versus 52 samples of HN using JMP Genomics 6.1 of SAS identified a single SNP present in BPIFA1 gene on chromosome number 13 of *B. indicus* at position 63251805 significantly associated with HC. The gene BPIFA1 is also known as PLUNC (Palate, Lung and Nasal Epithelium Carcinoma associated). It is specifically expressed in upper airways and nasopharyngeal regions of mammals (Iwao et al. 2001). The exact biological function of this gene is not known; however, it has been suggested to be involved in inflammatory responses to irritants in the upper airways (Liu et al. 2013). It was suggested as a potential molecular marker for detection of micrometastasis in non-small-cell lung cancer by many groups of researchers (Benedikova et al. 2012; Iwao et al. 2001; Li et al. 2014; Yew et al. 2012). PLUNC1 regulates cell proliferation, differentiation and apoptosis through miR-141, which in turn regulates PTEN and p27 expression. This signalling axis is negatively regulated by the EBV-coded gene LMP1. Thus, SPLUNC1 suppresses tumour formation and its inhibition by LMP1 provides a route for tumorigenesis (Chen et al. 2013).

The SNP identified in our study has already been reported as dBSNP ID rs136870681 in whole genome assembly of *B. taurus* cattle. BPI fold containing family A, member 1 (BPIFA1) gene is 7696 bp long gene with six exons in it [NCBI Reference Sequence: AC_000170.1; region from base 63247184 to 63254779]. A missense mutation at 63251805 position results in allele change TTC ⇒ TCC at 189 position amino acid substitution that resulted in F (Phe) ⇒ S (Ser) residual change (Zimin et al. 2009). In the mutation database 4 nsSNPs for the BPIFA1 gene has already been identified, but information regarding association study is not available. The PROVEAN classified this substitution mutation as a neutral in functional impact but, analysis based on I-Mutant 2.0 indicates that mutant protein has decreased stability at pH 7. Analysis of SCA resulted in low accessible surface area thought to be because of disrupt in ligand-binding site or protein binding sites that will affect protein function and alter the protein stability or folding rate (Ng and Henikoff 2006).

## Conclusions

We demonstrated an efficient approach for limited number of SNP discovery and validation in targeted genomics regions in a large number of samples combining PCR amplification and next-generation sequencing technologies. Using of the 91 SNPs reported in 69 aberrantly expressed genes in HC, we showed that this approach is effective in identification and validation of SNPs. We identified SNP [T → C] in chromosome No 13 at position 63251805 (dBSNP ID rs136870681) of BPIFA1 gene and shows association with event of HC which reflects it’s potential to be a genetic marker based on case control analysis. Further investigation is required to establish this SNP as a molecular marker of HC. Other mutations in associated genes and its pathways need to be further investigated. SNPs identified in this study will enrich dbSNP database of NCBI and will be useful resource for array designing, especially for *B. indicus* animals. The present findings would provide basis for further screening of genes and identification of markers for early diagnosis and therapeutic intervention of HC.

## Electronic supplementary material

Below is the link to the electronic supplementary material.

Supplementary material 1 (XLSX 64 kb)

## Abbreviations

HC: Horn cancer
HN: Horn normal
